# Analysis of Entropy Generation via Non-Similar Numerical Approach for Magnetohydrodynamics Casson Fluid Flow with Joule Heating

**DOI:** 10.3390/e26080702

**Published:** 2024-08-19

**Authors:** Hanen Louati, Sajid Khan, Muavia Mansoor, Shreefa O. Hilali, Ameni Gargouri

**Affiliations:** 1 Mathematics Department, Faculty of Science, Northern Border University, Arar 73213, Saudi Arabia; hanen.louati@nbu.edu.sa; 2 Department of Mechanical Engineering, University of Wah, Wah Cantt 47040, Pakistan; sajid.khan@wecuw.edu.pk; 3 Department of Mathematics, University of Wah, Wah Cantt 47040, Pakistan; 4 Department of Mathematics, College of Sciences and Arts (Majardah), King Khalid University, Magardah 61937, Saudi Arabia; shoamin@kku.edu.sa; 5 Mathematics Department, College of Humanities and Science in Al Aflaj, Prince Sattam Bin Abdulaziz University, Alkharj 11912, Saudi Arabia; a.gargouri@psau.edu.sa

**Keywords:** entropy generation, Casson fluid, thermal radiations, stagnation point, chemical reaction

## Abstract

This analysis emphasizes the significance of radiation and chemical reaction effects on the boundary layer flow (BLF) of Casson liquid over a linearly elongating surface, as well as the properties of momentum, entropy production, species, and thermal dispersion. The mass diffusion coefficient and temperature-dependent models of thermal conductivity and species are used to provide thermal transportation. Nonlinear partial differential equations (NPDEs) that go against the conservation laws of mass, momentum, heat, and species transportation are the form arising problems take on. A set of coupled dimensionless partial differential equations (PDEs) are obtained from a set of convective differential equations by applying the proper non-similar transformations. Local non-similarity approaches provide an analytical approximation of the dimensionless non-similar system up to two degrees of truncations. The built-in Matlab (Version: 7.10.0.499 (R2010a)) solver bvp4c is used to perform numerical simulations of the local non-similar (LNS) truncations.

## 1. Introduction

One kind of non-Newtonian fluid that falls within the category of shear thinning liquids that induce yield stress is Casson fluid. On the other hand, if shear stress is greater than yield stress, it begins to move. Rouleaux are a type of chainlike structure that can be produced in aqueous base plasma due to the presence of certain materials such as fibrinogen, protein, globulin, and human red blood cells. Several investigations have previously been conducted to identify the Casson fluid across flat or cylindrical stretched sheets in various physical conditions. Jelly, toothpaste, concentrated fruit juices, tomato soup, honey, shampoo, blood, etc., are a few examples of Casson fluids [1]. This model has been investigated and examined by numerous scientists and researchers under various rheological circumstances. Mernone et al. [2], for example, looked into the peristaltic transfer of a Casson fluid. Blood flow is explained by the Casson fluid model, which is quite useful for simulating blood hemodialysis and oxygenators. Raju et al. [3] explained the impact of mass transport and heat in Casson fluid flow on the porous stretched surface. Regarding thermally corrugated porous enclosures with Casson liquid suspension, K. U. Rehman et al. have investigated finite element thermal analysis [4]. Hussain et al. provided an investigation of the Casson rheological fluid containing gold nanoparticles under the effect of magnetic and gravitational forces [5]. Nazir et al. propose a theoretical framework for the mathematical modeling of the Casson rheological fluid [6]. Using the shooting technique, Kempannagari et al. [7] have addressed the MHD stagnation-point flow of micropolar fluid over a curved surface under the effects of radiation, Joule heating, and changing thermal conductivity. Runge–Kutta–Fehlberg solutions have been employed by Gopal et al. [8] to describe the effects of MHD and Joule heating on the Casson fluid flow across the stretching sheet. Muthtamilselvan et al. [9] discussed the stagnation-point flow of the Williamson nanofluid containing gyrotactic microorganisms. The results revealed that the density of microorganisms reduces with the stretching velocity.

Radiation effects have significant applications in physics and engineering processes. It is important for processes involving hot materials or liquids and space technology, where the effects of flow temperature are studied for various flows. In the polymer processing business, thermal radiation effects may be crucial in managing heat transmission. Heat transmission via radiation is a phenomenon known as thermal radiation. Its influence on MHD [10,11,12] flows has significance in engineering and industry, involving high thermal effects in the production of paper plates, petroleum pumps, electronic chips, and metallic item cooling. Kumar et al. [13] utilize the nonlinear thermal radiation influence to study MHD shear-thickening fluid dynamics via stretchy geometry.

A point of stagnation is reached when velocity equals zero. It depicts the flow of fluid in a solid surface’s direct zone. As the fluid gets closer to the solid surface, it splits into two streams. Everywhere on the solid surface, the fluid is stationary because of the no-slip condition. N. Khan and Y.M. Chu [14] addressed modeling and dual solutions for magnetized mixed convective stagnation-point flow of a higher convected Maxwell fluid model with second-order velocity slip. The publications (see Refs. [15,16,17,18,19]) contain a thorough description of the stagnation-point flow across either a stretching or contracting sheet. Mahapatra and Gupta [20] investigated the MHD stagnation-point flow past a stretching sheet.

In the field of engineering, minimizing entropy generation is an emerging thermodynamic technique that is crucial to the design of contemporary heat management systems. Bejan [21] is credited with introducing the idea of entropy creation. He came to the conclusion that the process of minimizing entropy formation improves the system’s thermodynamic efficiency and increases the efficiency of a thermal system. Analyzing entropy in Casson fluid flow is essential for understanding various industrial and biological processes involving non-Newtonian fluids. The Casson fluid model is often applied to fluids with yield stress, such as blood flow in arteries [22], as well as in polymer processing and lubrication systems [23]. Conducting entropy analysis in these contexts is crucial for optimizing lubricant performance and extending the operational life of machinery. Additionally, Casson fluids can be utilized in cooling systems for electronic devices, where entropy generation analysis plays a key role in reducing energy loss and enhancing heat removal efficiency. This, in turn, is vital for improving the performance and durability of electronic components. The physical parameters such as Prandtl number and Schmidt number that significantly influence the analysis of entropy generation fall within the appropriate ranges, as documented in [24,25].

A heightened concentration boundary layer was observed for big activation energies, while the contrary was observed for increasing reaction rates, according to the results of a numerical analysis of the mathematical system using the shooting technique. Additionally, entropy generation for Williamson fluid was higher than for Newtonian fluid. Heat transmission of a mixed convection ferrofluid down a vertical duct with variable wall temperatures has been examined by Gul et al. [26]. They discovered that the primary factors influencing the various velocity and temperature outcomes are viscosity and thermal conductivity. The unstable electrically conducting Casson fluid flows across an oscillating vertical porous plate with constant wall temperature have been studied by Khalid et al. [27]. Gul et al. [28] studied the mixed convection time-dependent MHD flow of an incompressible nanofluid (four distinct forms) under three different flow conditions inside a channel filled with saturated porous medium with non-uniform wall temperatures. Entropy analysis plays a key role in enhancing the efficiency of various real-world devices and systems. For instance, in artificial heart valves, stents, and blood pumps, entropy analysis aids in optimizing design to minimize energy losses, improve blood circulation, and reduce the risk of clot formation. In lubrication systems, such as bearings, gears, and hydraulics, it can lead to the development of better lubricant formulations and more efficient system designs, thereby reducing frictional losses and prolonging the life of mechanical components. Additionally, optimizing the flow of Casson fluids in cooling systems, including heat sinks, liquid cooling systems, and thermal management units, through entropy analysis can improve heat dissipation, reduce energy consumption, and enhance the performance and longevity of electronic components.

The entropy number alone cannot adequately distinguish between different irreversibility mechanisms. To address this limitation, the Bejan number (Be) is introduced. The Bejan number is calculated by dividing the heat transfer irreversibility by the total entropy production. Its value ranges between 0 and 1: when Be equals 1, heat transfer irreversibility is dominant, whereas when Be equals 0, the irreversibility due to viscous dissipation prevails.

## 2. Problem Formulations

**Figure 1:**
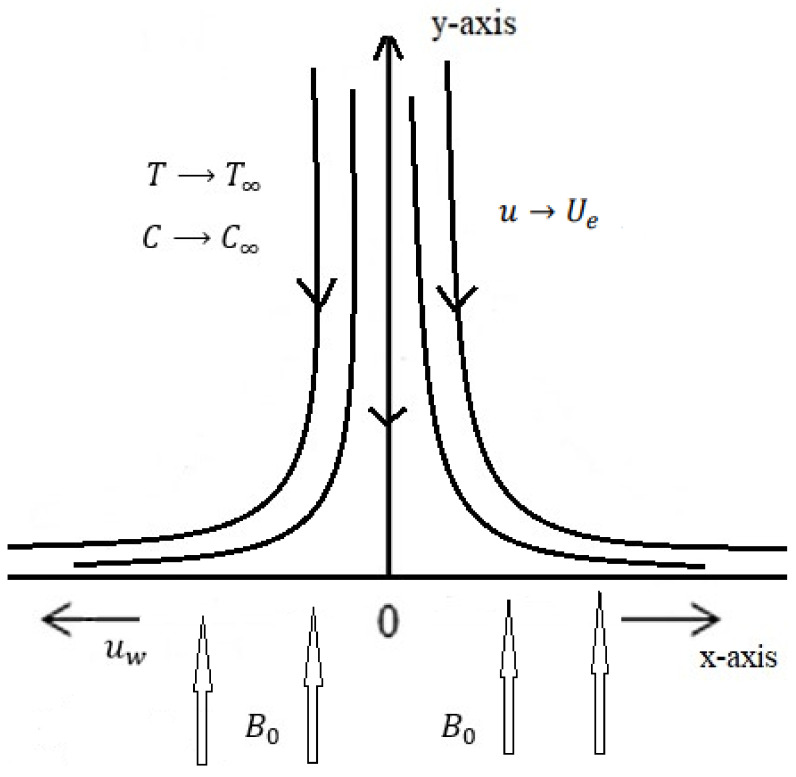
Geometry of the problem.

As seen in Figure 1, consider a flat vertical plate that has been adjusted parallel to a stationary fluid. The “x” and “y” coordinates represent the streamwise and crosswise directions, respectively. The surface expands at a linear rate, which causes the magnetized vertical flow to propagate. The “x”-momentum equation incorporates the linear buoyancy with respect to concentration and temperature variations. In addition, the energy equation takes thermal radiation and chemical reactions into account. The concentration equation makes the assumption that the chemical reaction is linear.

(1) $$u_{x}+v_{y}=0,$$

(2) $$uu_{x}+vu_{y}=U_{e}\frac{dU_{e}}{dx}+\nu \left(1+\frac{1}{\beta }\right)u_{yy}+g\beta_{T}\left(T-T_{\infty }\right)+g\beta_{C}\left(C-C_{\infty }\right)-\frac{\sigma {B_{0}}^{2}}{\rho }u,$$

(3) $$\left(uT_{x}+vT_{y}\right)=\alpha \left(T_{yy}\right)-\frac{1}{{\rho C}_{p}}{\left(q_{r}\right)}_{y}+\frac{\sigma {B_{0}}^{2}}{{\rho C}_{p}}u^{2}$$

(4) $$\left(uC_{x}+vC_{y}\right)=DC_{yy}-k_{1}\left(C-C_{\infty }\right).$$

The associated conditions are as follows:

(5) $$\left.\begin{array}{c} u=U_{w}\left(x\right),\;\;\;\;v=0,\;\;\;\;\frac{\partial T}{\partial y}=-\frac{h_{f}}{k_{f}}(T_{w}-T),\;C=C_{w}\;at\;y=0,\\ T\to T_{\infty },\;\;\;\;\;\;u\to U_{e},\;\;\;\;C\to C_{\infty }\;as\;\;y\to \infty . \end{array}\right\}$$

With the help of Rosseland estimation, the expression for $q_{r}$ is given by

(6) $$q_{r}=-\frac{4\sigma^{*}}{3k^{*}}T_{y}^{4}.$$

Ignoring $T^{4}$ and all higher terms, the expression may be expressed as

(7) $$T^{4}≅4T_{\infty }^{3}T-3T_{\infty }^{4}.$$

By using (6) and (7) in (4), we obtain

(8) $$\left(uT_{x}+vT_{y}\right)=\left(\alpha +\frac{16\sigma^{*}T_{\infty }^{3}}{3k^{*}\rho C_{p}}\right)\left(T_{yy}\right)$$

u and v are the component of velocity in the x and y directions. Uwx=uwexl is the velocity at the wall, Ue=ueexl is the velocity of the fluid away from the plate at free stream, β is the Casson parameter, ${B_{0}}^{2}$ is the magnetic field parameter, $C_{p}$ is the specific heat capacity, and $q_{r}$ is the radiative heat flux.

### 2.1. Non-Similar Transformation

The transformation of local non-similarity was first presented in 1970 by Sparrow et al. [29]. Two independent variables are involved in this transformation: one is non-similar and the other is pseudo-similar. By using this method, problems become dimensionless and partial differential equations remain partial differential equations. The local non-similarity method yields highly accurate results at all streamwise locations, except near the point of separation.

(9) $$\xi =e^{\frac{x}{l}},\eta =y\sqrt{\frac{U_{e}}{2l\upsilon }},\psi \left(x,y\right)=\sqrt{2\upsilon lU_{e}}f(\xi ,\eta ),u=U_{e}\frac{\partial f}{\partial \eta },\;v=-\sqrt{\frac{\upsilon U_{e}}{2l}}f-\sqrt{\frac{2\upsilon U_{e}}{l}}\xi \frac{\partial f}{\partial \xi }-\frac{yU_{e}}{2l}\frac{\partial f}{\partial \eta }f,\;U_{e}=u_{e}e^{\frac{x}{l}}\;\mathrm{and}\;\theta \left(\xi ,\eta \right)=\frac{\left(T-T_{\infty }\right)}{\left(T_{w}-T_{\infty }\right)},\varphi \left(\xi ,\eta \right)=\frac{\left(C-C_{\infty }\right)}{\left(C_{w}-C_{\infty }\right)}.$$

After applying non-similarity transformations on Equations (2), (4), and (8), they are reduced to (10)–(12):

(10) $${\left(\frac{\partial f}{\partial \eta }\right)}^{2}+\xi \frac{\partial^{2}f}{\partial \xi \partial \eta }\frac{\partial f}{\partial \eta }-\frac{f}{2}\frac{\partial^{2}f}{\partial \eta^{2}}-\xi \frac{\partial f}{\partial \xi }\frac{\partial^{2}f}{\partial \eta^{2}}=\left(1+\frac{1}{\beta }\right)\frac{\partial^{3}f}{\partial \eta^{3}}+\left\{\frac{{Gr}_{l}}{{R_{e}}^{2}}\theta +\frac{{Gr}_{c}}{{R_{e}}^{2}}\varphi \right\}-M\frac{\partial f}{\partial \eta },$$

(11) $$Pr\left(f\frac{\partial \theta }{\partial \eta }-2\xi \frac{\partial \theta }{\partial \xi }\frac{\partial f}{\partial \eta }+2\xi \frac{\partial f}{\partial \xi }\frac{\partial \theta }{\partial \eta }+J{\left(\frac{\partial f}{\partial \eta }\right)}^{2}\right)=-\left(1+\frac{4R_{d}}{3}\right)\left(\frac{\partial^{2}\theta }{{\partial \eta }^{2}}\right),$$

(12) $$\left(f\frac{\partial \varphi }{\partial \eta }-2\xi \frac{\partial \varphi }{\partial \xi }\frac{\partial f}{\partial \eta }+2\xi \frac{\partial f}{\partial \xi }\frac{\partial \varphi }{\partial \eta }\right)=-\frac{1}{2Sc}\frac{\partial^{2}\varphi }{{\partial \eta }^{2}}+\gamma \varphi .$$

Associated conditions are

(13) $$\left.\begin{array}{c} f^{\prime }\left(\xi ,\eta \right)=\epsilon ,\;\;\;\;\theta^{\prime }\left(\xi ,\eta \right)=-\sqrt{\frac{2}{Re}}Bi\left(1-\theta \right),\;\;\;\;f\left(\xi ,\eta \right)=\xi \frac{\partial f}{\partial \xi },\varphi \left(\xi ,\eta \right)=1\;at\;\eta =0,\\ \theta \left(\xi ,\eta \right)\to 0,\;f^{\prime }\left(\xi ,\eta \right)\to 1,\;\varphi \left(\xi ,\eta \right)\to 0\;at\;\eta ⟶\infty . \end{array}\right\}$$

${Gr}_{l}=\frac{g\beta_{T}\left(T_{f}-T_{\infty }\right)l^{3}}{v^{2}},{Gr}_{C}=\frac{g\beta_{C}\left(C_{w}-C_{\infty }\right)l^{3}}{v^{2}}$ is the Grashof number for heat and mass transfer, $R_{e}=U_{e}\frac{l}{\upsilon }$ is the Reynolds number, $Bi=\frac{h_{f}}{k_{f}}l$ is the Biot number, and $\epsilon =\frac{U_{w}}{U_{e}}$ is the ratio of stretching to free stream velocities.

### 2.2. Non-Similarity Technique

For the first and second level of truncation, the following equations are used.

#### 2.2.1. Truncation Level 1

We consider $\xi \frac{\partial (.)}{\partial \xi }=0$ at this level to obtain the required ordinary differential equations:

(14) $$f^{\prime \prime \prime }=\frac{1}{\left(1+\frac{1}{\beta }\right)}\left({f^{\prime }}^{2}-\frac{f}{2}f^{\prime \prime }-\frac{{Gr}_{l}}{{R_{e}}^{2}}\theta -\frac{{Gr}_{c}}{{R_{e}}^{2}}\varphi +Mf^{\prime }\right),$$

(15) $$\theta^{\prime \prime }=\frac{-Pr}{\left(1+\frac{4R_{d}}{3}\right)}\left\{f\theta^{\prime }+J({f^{\prime })}^{2}\right\}$$

(16) $$\varphi^{\prime \prime }=2Sc\left(\gamma \varphi -f\varphi^{\prime }\right)$$

Associated conditions are

(17) $$\left.\begin{array}{c} f^{\prime }\left(\xi ,\eta \right)=\epsilon ,\;\;\;\theta^{\prime }\left(\xi ,\eta \right)=-\sqrt{\frac{2}{Re}}Bi\left(1-\theta \right),\;\;\;\;\;\;\;\;f\left(\xi ,\eta \right)=0,\;\varphi \left(\xi ,\eta \right)=1\;at\;\eta =0\\ \theta \left(\xi ,\eta \right)\to 0,\;f^{\prime }\left(\xi ,\eta \right)\to 1,\;\varphi \left(\xi ,\eta \right)\to 0\;at\;\eta ⟶\infty .\; \end{array}\right\}$$

#### 2.2.2. Truncation Level 2

By introducing the new variables, the derivative of the following functions will be written as

(18) $$\mathrm{Let}\;\frac{\partial f}{\partial \xi }=p,\;\frac{\partial^{2}f}{\partial \xi \partial \eta }=\frac{\partial f^{\prime }}{\partial \xi }=p^{\prime },\frac{\partial \theta }{\partial \xi }=q,\frac{\partial^{2}\theta }{\partial \xi \partial \eta }=\frac{\partial \theta^{\prime }}{\partial \xi }=q^{\prime },\frac{\partial \varphi }{\partial \xi }=g,\frac{\partial^{2}\varphi }{\partial \xi \partial \eta }=\frac{\partial \varphi^{\prime }}{\partial \xi }=g^{\prime }\;f^{\prime \prime \prime }=\frac{1}{\left(1+\frac{1}{\beta }\right)}\left({f^{\prime }}^{2}+\xi p^{\prime }f^{\prime }-\frac{f}{2}f^{\prime \prime }-\xi pf^{\prime \prime }-\frac{{Gr}_{l}}{{R_{e}}^{2}}\theta -\frac{{Gr}_{c}}{{R_{e}}^{2}}\varphi +Mf^{\prime }\right),$$

(19) $$\theta^{\prime \prime }=\frac{Pr}{\left(1+\frac{4R_{d}}{3}\right)}\left(2\xi qf^{\prime }-f\theta^{\prime }-2\xi p\theta^{\prime }f\theta^{\prime }-J({f^{\prime })}^{2}\right),$$

(20) $$\varphi^{\prime \prime }=2Sc\left(2\xi \;gf^{\prime }-f\varphi^{\prime }-2\xi p\varphi^{\prime }+\gamma \varphi \right).$$

With conditions,

(21) $$\left.\begin{array}{c} f^{\prime }\left(\xi ,\eta \right)=\epsilon ,\;\;\;\;\;\;\theta^{\prime }\left(\xi ,\eta \right)=-\sqrt{\frac{2}{Re}}Bi\left(1-\theta \right),\;\;f\left(\xi ,\eta \right)=0,\;\;\;\;\;\varphi \left(\xi ,\eta \right)=1\;at\;\eta =0,\\ \;\;\;\;\;\theta \left(\xi ,\eta \right)\to 0,\;\;\;\;\;f^{\prime }\left(\xi ,\eta \right)\to 1,\;\;\;\;\;\varphi \left(\xi ,\eta \right)\to 0\;at\;\eta ⟶\infty . \end{array}\right\}$$

Taking the derivative with respect to ξ for (18)–(20), we obtain

(22) $$p^{\prime \prime \prime }=\frac{1}{\left(1+\frac{1}{\beta }\right)}\left(3p^{\prime }f^{\prime }+\xi {p^{\prime }}^{2}f^{\prime }-\frac{3}{2}pf^{\prime \prime }-\frac{f}{2}p^{\prime \prime }-\xi pp^{\prime \prime }-\frac{{Gr}_{l}}{{R_{e}}^{2}}q-\frac{{Gr}_{c}}{{R_{e}}^{2}}g+Mp^{\prime }\right),$$

(23) $$q^{\prime \prime }=\frac{Pr}{\left(1+\frac{4R_{d}}{3}\right)}\left(2\xi qp^{\prime }-3p\theta^{\prime }-fq^{\prime }+2qf^{\prime }-2\xi pq^{\prime }-2Jf^{\prime }p\right),$$

(24) $$g^{\prime \prime }=2Sc\left(\gamma g-3p\varphi^{\prime }-fg^{\prime }+2gf^{\prime }+2\xi gp^{\prime }-2\xi pg^{\prime }-\xi \gamma \varphi \right).$$

With conditions,

(25) $$\left.\begin{array}{c} p^{\prime }\left(\xi ,\eta \right)=0,\;\;\;\;q^{\prime }\left(\xi ,\eta \right)=\sqrt{\frac{2}{Re}}Bi.q,\;\;\;\;p\left(\xi ,\eta \right)=0,\;\;\;\;g\left(\xi ,\eta \right)=0\;at\;\eta =0,\\ q\left(\xi ,\eta \right)\to 0,\;\;\;\;p^{\prime }\left(\xi ,\eta \right)\to 0,\;\;\;\;g\left(\xi ,\eta \right)\to 0\;at\;\eta ⟶\infty . \end{array}\right\}$$

The mathematical expression of physical interest is specified by

(26) $$C_{f}=\frac{\tau_{w}}{\rho U_{e}^{2}},Nu_{x}=\frac{xq_{w}}{k\left(T_{w}-T_{\infty }\right)},Sh_{x}=\frac{xq_{m}}{D_{B}\left(C_{w}-C_{\infty }\right)}$$

$\tau_{w}=\mu {\left(\frac{\partial u}{\partial y}\right)}_{y=0},$ $q_{w}=\left(k+\frac{16\sigma^{*}T_{\infty }^{3}}{3k^{*}}\right){\left(\frac{\partial T}{\partial y}\right)}_{y=0},$ and $q_{m}=-D{\left(\frac{\partial C}{\partial y}\right)}_{y=0}$ are the shear stress, the wall heat flux, and wall mass flux, respectively.

The significant transport quantities [30] are given as

(27) $${2^{-\frac{1}{2}}Re}_{x}^{\frac{1}{2}}C_{fx}=\left(1+\frac{1}{\beta }\right)\frac{1}{\epsilon^{2}}{\left(\frac{\partial^{2}f}{\partial \eta^{2}}\right)}_{\eta =0}$$

(28) $$\sqrt{2}{Re}_{x}^{-\frac{1}{2}}{Nu}_{x}=-\left(1+\frac{4}{3}R_{d}\right){\left(\frac{\partial \theta }{\partial \eta }\right)}_{\eta =0}$$

(29) $$\sqrt{2}{Re}_{x}^{-\frac{1}{2}}{Sh}_{x}=-{\left(\frac{\partial \varphi }{\partial \eta }\right)}_{\eta =0}$$

### 2.3. Entropy Analysis

The loss of productive work in an engineering system is directly correlated with the generation of entropy. According to But et al. [31], the volumetric entropy expression for a viscous incompressible Casson fluid in the Cartesian coordinate system is as follows:

(30) $$S_{G}=\frac{\kappa }{T_{\infty }^{2}}\;\left(1+\frac{16\sigma_{1}T_{\infty }^{3}}{3k^{*}k}\right){\left(\frac{\partial T}{\partial y}\right)}^{2}+\frac{\mu }{T_{\infty }}\;\;\left(1+\frac{1}{\beta }\right){\left(\frac{\partial u}{\partial y}\right)}^{2}+\frac{D}{C_{\infty }}{\left(\frac{\partial C}{\partial y}\right)}^{2}+\frac{D}{T_{\infty }}\left(\frac{\partial T}{\partial y}\right)\left(\frac{\partial C}{\partial y}\right)+\frac{\sigma {B_{0}}^{2}}{T_{\infty }}u$$

(31) $$S_{G}=\frac{\kappa }{T_{\infty }^{2}}\;\left(1+\frac{16\sigma_{1}T_{\infty }^{3}}{3k^{*}k}\right){\left(T_{w}-T_{\infty }\right)}^{2}\frac{U_{e}}{2l\upsilon }{\theta^{\prime }}^{2}+\frac{\mu }{T_{\infty }}\;\;\left(1+\frac{1}{\beta }\right)\left(\frac{{U_{e}}^{3}}{2lv}{f^{\prime \prime }}^{2}\right)\;\;+\frac{D_{B}}{C_{\infty }}{\left(C_{w}-C_{\infty }\right)}^{2}\frac{U_{e}}{2l\upsilon }{\varphi^{\prime }}^{2}+\frac{D_{B}}{T_{\infty }}\left(T_{w}-T_{\infty }\right)\left(C_{w}-C_{\infty }\right)\frac{U_{e}}{2l\upsilon }\theta^{\prime }\varphi^{\prime }+\frac{\sigma }{T_{\infty }}{B_{0}}^{2}{U_{0}}^{2}{f^{\prime }}^{2}$$

The irreversibility of heat transfer causes the first part of the statement above to arise; viscous dissipation causes the second part to appear; and the concentration of nanoparticles causes the third component to generate local entropy. Using Equation (9) in the above equations yields the entropy number expression in a dimensionless form:

(32) $$N_{s}=\frac{S_{G}}{S_{G_{0}}}={Re}_{l}^{\frac{1}{2}}\left(1+\frac{4}{3}R_{d}\right)\theta^{\prime 2}+\frac{Br}{\;\Omega_{T}}\frac{{Re}_{l}}{2}\left(1+\frac{1}{\beta }\right)f^{\prime \prime 2}+\epsilon \left(\frac{\Omega_{C}}{\;\Omega_{T}}\right)\theta^{\prime }\varphi^{\prime }+\epsilon {\frac{{Re}_{l}}{2}\left(\frac{\Omega_{C}}{\;\Omega_{T}}\right)}^{2}\varphi^{\prime 2}+M\frac{Br}{\;\Omega_{T}}R_{el}{f^{\prime }}^{2}$$

where $R_{d}=\frac{4\sigma_{1}T_{\infty }^{3}}{k^{*}k},$ $\Omega_{T}=\frac{∆T}{T_{\infty }},$ $\Omega_{C}=\frac{∆C}{C_{\infty }},$ $Br=\mu {U_{e}}^{2}/\kappa ∆T,$ $M=\frac{\sigma {B_{0}}^{2}l}{\rho U_{e}}$ and $\epsilon =RD_{B}C_{\infty }/\kappa$ are the characteristic entropy, the dimensionless temperature, concentration differences, the Brinkman number, and the nanoparticle mass transfer parameter, respectively. Classifying the dominance of the irreversibility mechanism is not possible with the entropy number. The Bejan number is created to examine the irreversibility method in order to solve this problem. The formation of the Bejan number Be involves dividing the irreversibility of heat transfer by the overall creation of entropy. The Bejan number (Be) for the current analysis has the following form (see, for instance, [32]):

(33) $$Be=\frac{{Re}_{l}^{\frac{1}{2}}\left(1+\frac{4}{3}R_{d}\right)\theta^{\prime 2}+\epsilon \left(\frac{\Omega_{C}}{\;\Omega_{T}}\right)\theta^{\prime }\varphi^{\prime }+\epsilon {\frac{{Re}_{l}}{2}\left(\frac{\Omega_{C}}{\;\Omega_{T}}\right)}^{2}\varphi^{\prime 2}}{{Re}_{l}^{\frac{1}{2}}\left(1+\frac{4}{3}R_{d}\right)\theta^{\prime 2}+\frac{Br}{\;\Omega_{T}}\frac{{Re}_{l}}{2}\left(1+\frac{1}{\beta }\right)f^{\prime \prime 2}+\epsilon \left(\frac{\Omega_{C}}{\;\Omega_{T}}\right)\theta^{\prime }\varphi^{\prime }+\epsilon {\frac{{Re}_{l}}{2}\left(\frac{\Omega_{C}}{\;\Omega_{T}}\right)}^{2}\varphi^{\prime 2}+M\frac{Br}{\;\Omega_{T}}{Re}_{l}{f^{\prime }}^{2}}.$$

The aforementioned equation unequivocally demonstrates that the range of Be is between 0 and 1: at Be=1, the irreversibility mechanism resulting from viscous dissipation and heat mass diffusion dominates the entropy generation; at Be=1/2, the involvement in entropy production due to heat transfer and the sum of viscous dissipation and heat and mass diffusion are equal; and at Be=0, the entropy production is dominated by heat transfer.

### 2.4. Solution Procedure

A numerical scheme called bvp4c is taken into consideration for solving ordinary differential equations that are derived by applying transformations on the governing equations of the specified flow issue. The formula known as three-stage Lobatto III, which yields fourth-order accuracy when solving a system of ordinary differential equations (ODEs), serves as the foundation for the solver. First-order ordinary differential equations (ODEs), boundary data, and an initial guess are required by the solver. We receive a system of initial value issues for the solver to solve if the differential equations have more than one order.

$$f^{\prime }=f_{2},\;f^{\prime \prime }=f_{3}$$

$${f_{3}}^{\prime }=f^{\prime \prime \prime }=1/\left(1+1/\beta \right)\left({f_{2}}^{2}+\xi f_{9}f_{2}-\frac{f_{1}}{2}f_{3}-\xi f_{8}f_{3}-\frac{{Gr}_{l}}{{R_{e}}^{2}}f_{4}-\frac{{Gr}_{c}}{{R_{e}}^{2}}f_{6}+Mf_{2}\right),$$

$$\theta^{\prime }=f_{5},$$

$$\;\;\;\;\;\;\;\;\;\;\;\;\;\;\;\;\;\;\;\;\;\;\;\;\;\;\;\;\;\;\;\;\;\;\;\;\;\;\;\;\;\;\;\;\;\;\;\;{f_{5}}^{\prime }=\theta^{\prime \prime }=Pr/(1+4R_{d}/3)\left(2\xi \;f_{11}f_{2}-f_{1}f_{5}-2\xi f_{8}f_{7}-J{f_{2}}^{2}\right),$$

$$\varphi^{\prime }=f_{7},$$

$$\;\;\;\;\;\;\;\;\;\;\;\;\;\;\;\;\;\;\;\;\;\;\;\;\;\;\;\;\;\;\;\;\;\;\;\;\;\;\;\;\;\;\;\;\;\;\;\;\;\;\;\;\;\;\;\;\;{f_{7}}^{\prime }=\varphi^{\prime \prime }=2Sc\left(2\xi \;f_{13}f_{2}-f_{1}f_{7}-2\xi f_{7}f_{8}+\gamma f_{6}\right),$$

$$p^{\prime }=f_{9},\;p^{\prime \prime }=f_{10},$$

$${f_{11}}^{\prime }=p^{\prime \prime \prime }=1/\left(1+1/\beta \right)\left(3f_{9}f_{2}+\xi {f_{9}}^{2}f_{2}-\frac{3}{2}f_{8}f_{3}-\frac{f_{1}}{2}f_{10}-\xi f_{8}f_{10}-\frac{{Gr}_{l}}{{R_{e}}^{2}}f_{11}-\frac{{Gr}_{c}}{{R_{e}}^{2}}f_{13}+Mf_{9}\right),$$

$$q^{\prime }=f_{12},$$

$${f_{13}}^{\prime }=q^{\prime \prime }=Pr/(1+4R_{d}/3)\left(2\xi f_{11}f_{9}-3f_{8}f_{5}-f_{1}f_{12}+2f_{11}f_{2}-2\xi f_{8}f_{12}-2Jf_{2}f_{8}\right),$$

$${\;\;\;\;\;\;\;\;\;\;\;\;\;\;\;\;\;\;\;\;\;\;\;\;\;\;\;\;\;\;\;\;\;\;\;\;\;\;\;\;\;\;\;\;\;\;\;\;\;\;\;\;\;\;\;\;\;\;\;\;\;\;\;\;\;\;\;\;\;\;\;\;\;\;\;\;\;\;\;\;\;\;\;g}^{\prime }=f_{14},$$

$${f_{15}}^{\prime }=g^{\prime \prime }=2Sc\left(\gamma f_{13}-3f_{8}f_{7}-f_{1}f_{14}+2f_{13}f_{2}+2\xi f_{13}f_{9}-2\xi f_{8}f_{14}-\xi \gamma f_{6}\right).$$

where $f=f_{1},\theta =f_{4},\varphi =f_{6},p=f_{8},q=f_{11}$ and $g=f_{13}$ and the related boundary data read as

$${\left[f_{1}0+2\xi f_{8},f_{2}0-\epsilon ,f_{2}inf-1,f_{5}0+\sqrt{\frac{2}{Re}}Bi\left(1-f_{4}\right),f_{4}inf,\;f_{6}0\;-1,\;f_{6}inf,\;f_{8}0+2f_{8},\;f_{9}0,\;f_{9}inf,\;f_{12}0\;-\sqrt{\frac{2}{Re}}Bif_{11},\;f_{11}inf,\;f_{13}0,\;f_{13}inf\right]}^{t}$$

The use of a non-similar numerical approach allows for the analysis of complex flow phenomena that do not conform to traditional similarity solutions. This method provides more flexibility and accuracy in solving partial differential equations governing the fluid flow. The non-similar numerical approach fills the gap left by traditional methods in terms of providing sufficient predictive capabilities for real-world problems that are complex. It makes it possible to accurately simulate intricate fluid behaviors in a variety of situations. 

## 3. Results and Discussions

**Figure 2:**
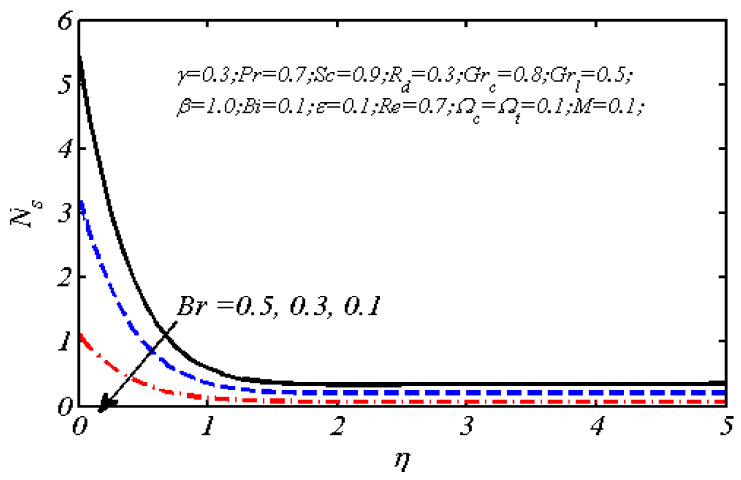
Local entropy generation profile for different *Br.*

**Figure 3:**
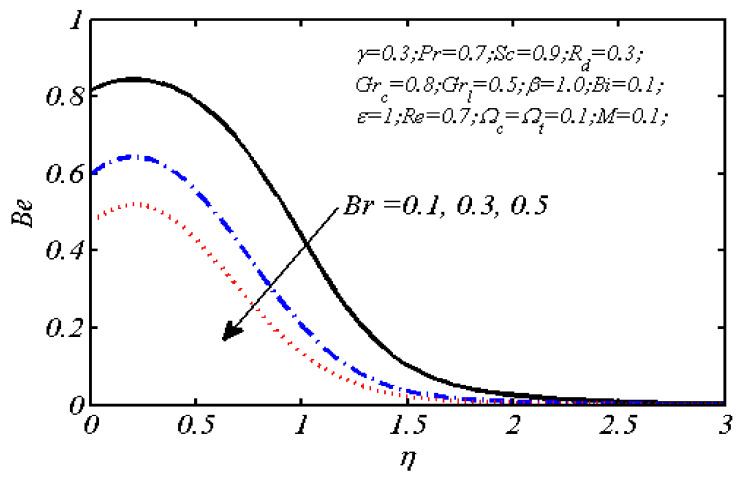
Be plotted against η for different Brinkman numbers.

**Figure 4:**
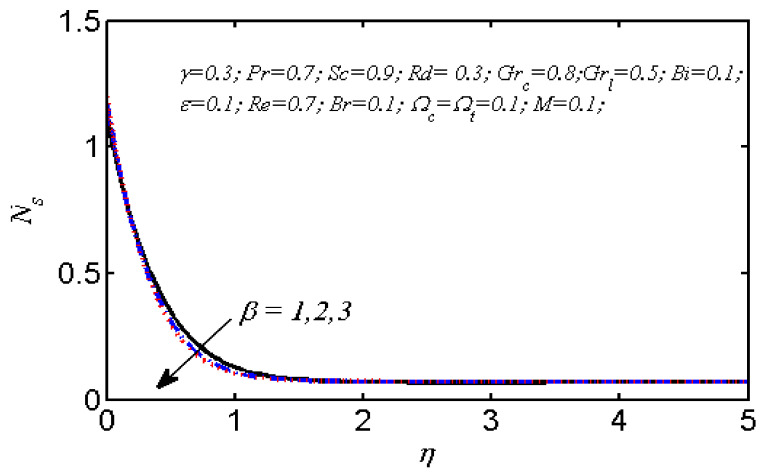
Dependence of local entropy generation number on β.

**Figure 5:**
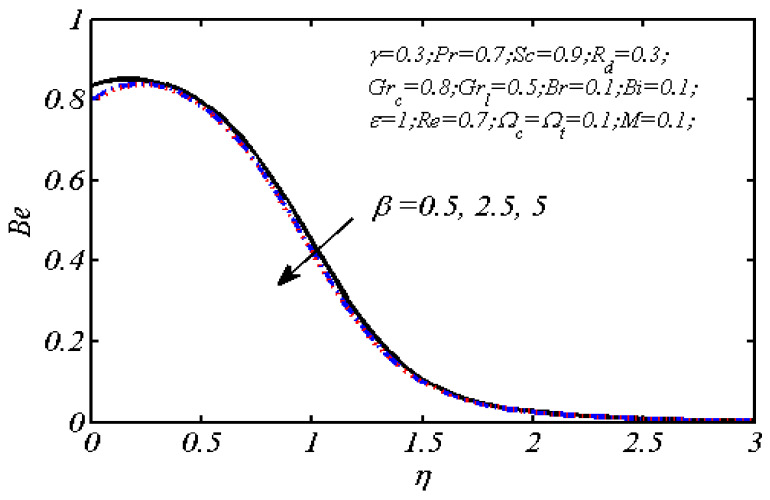
Impact of β on local Bejan number.

**Figure 6:**
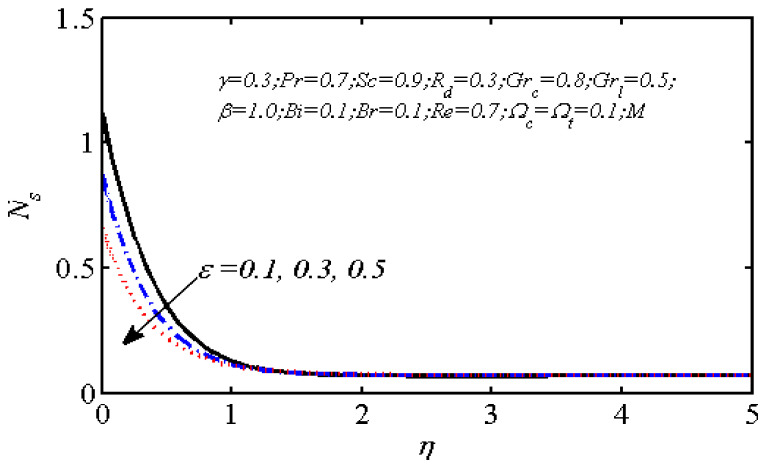
Variations in local entropy generation due to ε.

**Figure 7:**
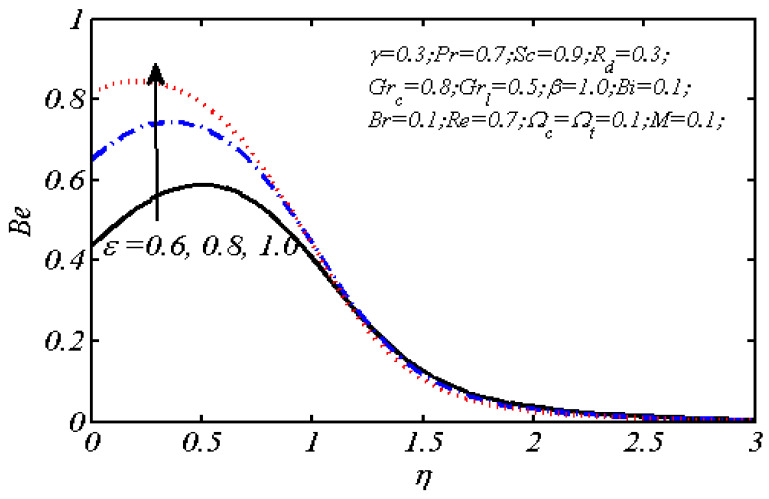
Local Bejan number plotted for different values of ε.

**Figure 8:**
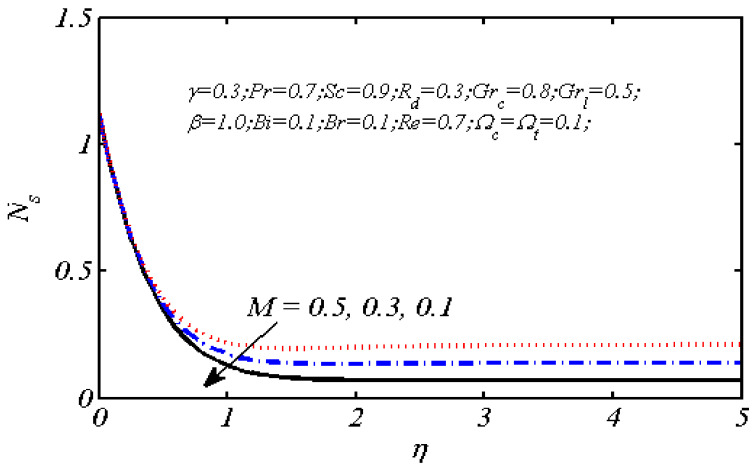
Effects of M on local entropy generation.

**Figure 9:**
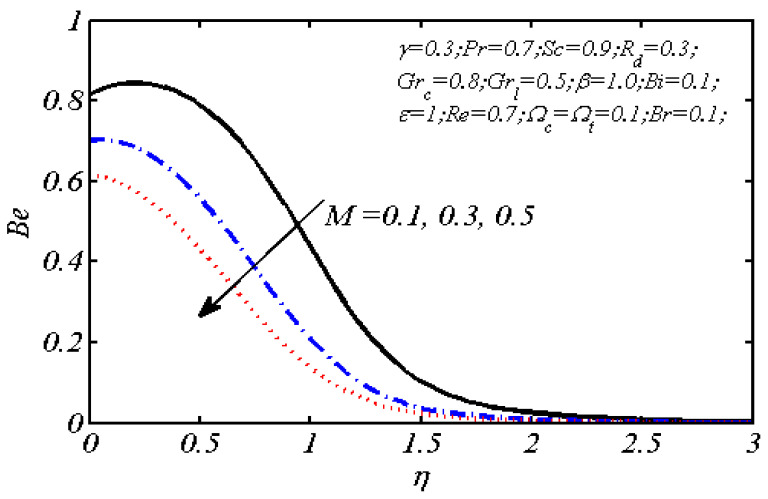
Local Bejan number profile for various values of *M.*

**Figure 10:**
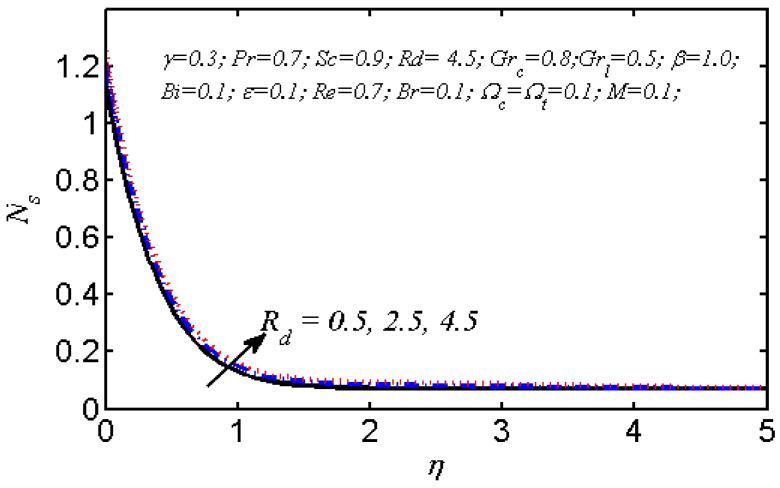
Local entropy generation profile for various values of $R_{d}$.

**Figure 11:**
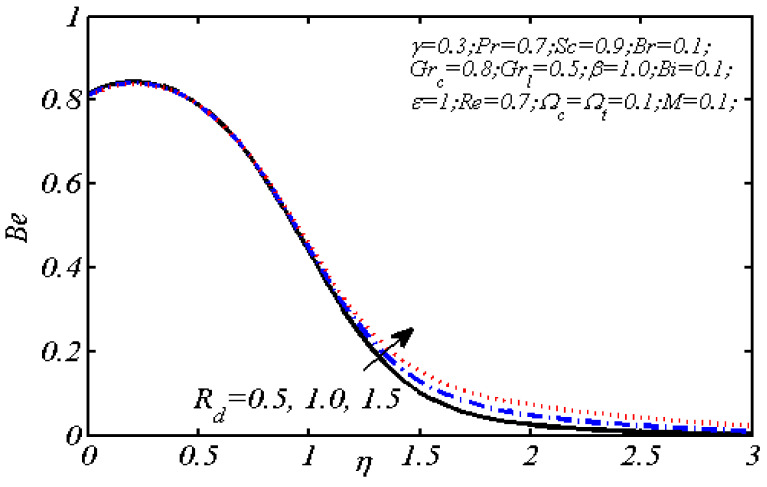
Influence of $R_{d}$ on local Bejan number profile.

**Figure 12:**
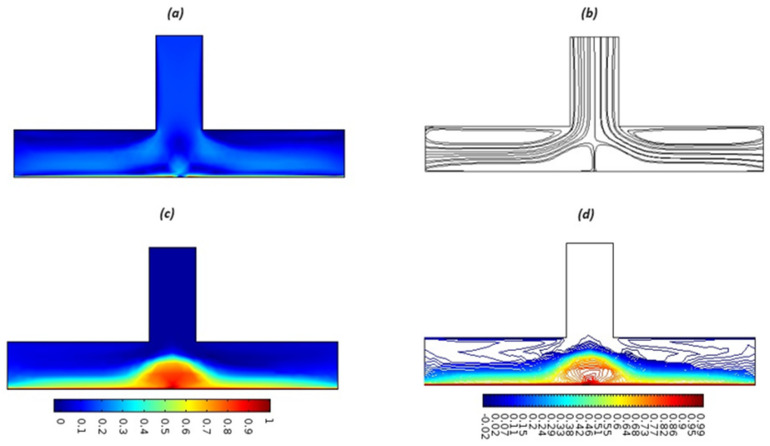
Surface and streamlines for $U_{e}=0.1$. (**a**) Surface plot of the velocity profile, (**b**) streamlines, (**c**) surface plot for the temperature profile (**d**) Isothermal contours.

**Figure 13:**
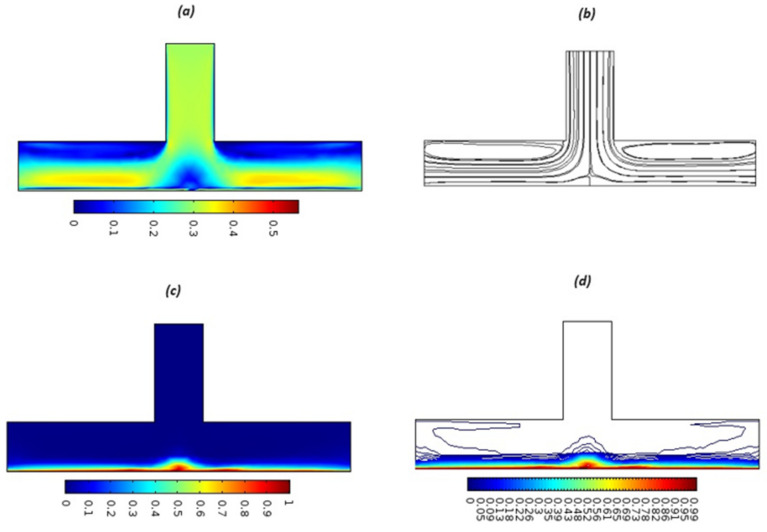
Surface and streamlines for $U_{e}=0.3$. (**a**) Surface plot of the velocity profile, (**b**) streamlines, (**c**) surface plot for the temperature profile (**d**) Isothermal contours.

**Figure 14:**
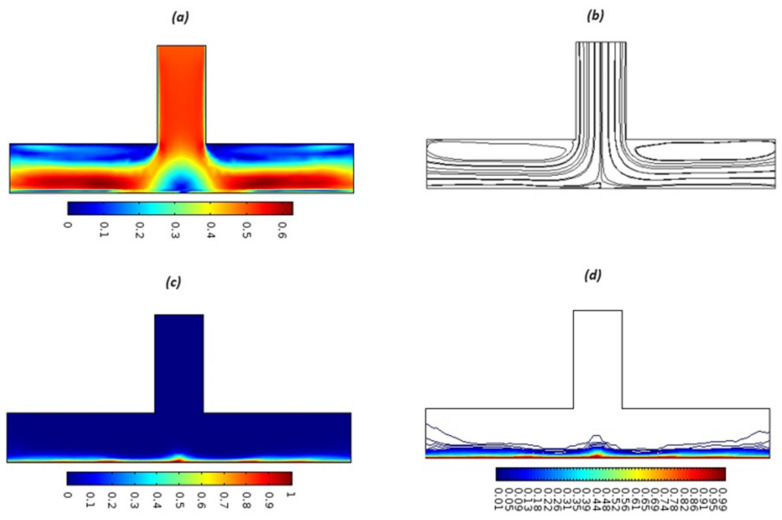
Surface and streamlines for $U_{e}=0.5$. (**a**) Surface plot of the velocity profile, (**b**) streamlines, (**c**) surface plot for the temperature profile (**d**) Isothermal contours.

Equations (18)–(20) and (22)–(24) with boundary conditions (21) and (25) were solved numerically using the built-in MATLAB package, bvp4c, to obtain a numerical solution. Table 1 presents the numerical values for the skin friction coefficient, local Nusselt number, and local Sherwood number. The data indicate that an increase in the Prandtl number and radiation parameter leads to an enhancement in the local Nusselt number. This is because the Prandtl number is inversely related to thermal diffusivity, which results in a decrease in thermal diffusivity. As a result, the temperature slows down, causing the thermal boundary layer to contract, and thereby increasing the local Nusselt number. The skin friction coefficient rises with an increase in the parameter β, which is due to the more rapid dispersion of momentum in non-Newtonian fluids compared to Newtonian fluids. This increased dispersion occurs because non-Newtonian fluids sustain a greater applied force than Newtonian fluids. Additionally, the local Sherwood number decreases with an increase in the Schmidt number, and the skin friction coefficient decreases as the Grashof number in heat transfer declines. Figure 2, Figure 3, Figure 4, Figure 5, Figure 6, Figure 7, Figure 8, Figure 9, Figure 10 and Figure 11 explore the effects of the parameters involved on the entropy generation rate and Bejan number. The graphical results indicate that the stretching plate surface is a significant contributor towards the production of entropy. Figure 2 examines the effects of the Brinkman number (Br) on local entropy generation. The figure shows that entropy generation rises as Br increases. This increase is clearly attributed to fluid friction, leading to an elevation in entropy production. In physical terms, a higher Brinkman number indicates that a greater portion of the mechanical energy is being converted into heat due to internal friction within the fluid and consequently decreases the performance of the thermal system. Figure 3 illustrates the impact of the Brinkman number on the local Bejan number $\left(Be\right)$. As the Brinkman number increases, the local Bejan number decreases. Elevated Br values enhance the viscous effects, ultimately resulting in a dominance of fluid friction irreversibility. However, for a fixed value of Br, this figure illustrates that the major contributors to entropy production are diffusivity and heat transfer at the plate, and in the distant region (far from the boundary), fluid friction and magnetic field entropy become stronger. Table 2 presents the quantitative results for the average entropy generation number ($\bar{N_{s}})$ and average Bejan number $\left(\bar{Be}\right)$ as the Brinkman number (Br) varies. The average entropy generation demonstrates an upward trend with increasing Br, while the Bejan number shows the opposite trend. Increasing Br from 0.1 to 1.1, it is observed that $\bar{N_{s}}$ is increased up to 10.43 times and $\bar{Be}$ decreases up to 3.60 times. Figure 4 explores the impact of the Casson parameter β on the entropy profile. The decreasing trend has been observed in the entropy profile due to the increasing value of β, but this depreciation is not so prominent, which can also be observed from Table 3 in the case of average entropy ($\bar{N_{s}})$. A similar trend can be seen in Figure 5 for the case of Bejan numbers for different β values. Increasing the value of the mass transfer parameter ε results in decreasing behavior in the entropy generation profile, as shown in Figure 6. On the other hand, in Figure 7, increasing the value of ε also increases the Bejan number, which indicates that thermal irreversibility and diffusion entropy profile are more dominant. The impact of the magnetic parameter M on the entropy profile is presented in Figure 8, which shows an increment in $N_{s}$. When M changes from 0.1 to 1.1, $\bar{N_{s}}$ augments 4.81 times (see in Table 4). The Lorentz force acts as a damping force on fluid motion, converting kinetic energy into thermal energy. This conversion increases the process’s irreversibility. Due to the strengthened magnetic field parameter M, the Bejan number profile decreases, which ultimately depicts the dominance of fluid friction and magnetic field entropy. Figure 10 reveals the influence of the radiation parameter $R_{d}$ on the entropy profile. It is seen that entropy increases as $R_{d}$ increases. Enhanced radiative heat transfer results in increased energy dissipation rates. Because radiation tends to be directional and can cause uneven temperature distributions, it amplifies the system irreversibly, thus boosting entropy generation. Table 5 also shows the same impact upon average entropy. The local Bejan number is not influenced by the radiation parameter $R_{d}$ in the vicinity of the plate, but in the main flow region, the Bejan profile is enhanced, as one can see in Figure 11. Figure 12, Figure 13 and Figure 14 were drawn using software that uses the finite element method. The two-dimensional region is used as the geometry of the problem. The left and right walls are considered as outlets, the lower wall is considered to be a moving wall, part of the top wall is considered with no-slip conditions, and the rest of the wall is considered to be an inlet with velocity along the y-axis. The mixed convection and MHD effects are incorporated with different velocities of the plate. The solution is displayed in different graphs shown in Figure 12, Figure 13 and Figure 14. The impact of the physical parameters Brinkman number, Casson parameter, and magnetic parameter on entropy generation and Bejan number follows a trend similar to that reported by Butt et al. [33].

## 4. Conclusions

This paper mathematically models the heat and mass transfer of a thermally radiative, chemically reactive mixed convection flow of Casson fluid over a stretching sheet. By applying the local non-similarity method, the governing equations of the flow phenomena are transformed into their dimensionless form. The resulting partial differential equations are transformed to an ordinary differential equation, and then solved numerically using the bvp4c built-in solver in MATLAB. The results obtained with different physical parameters are plotted and discussed. The outcomes of this study are as follows:

- The local entropy generation number $N_{s}$ is enhanced by increases in the Brinkman number, magnetic field, and radiation parameter.
- An increase in the value of the Casson parameter and mass transfer parameter causes the local entropy generation number to decrease in the vicinity of the convective stretching surface.
- The average entropy generation number ($\bar{N_{s}})$ increases up to 10.43 times as Br changes from 0.1 to 1.1, while the average Bejan number $\left(\bar{Be}\right)$ decreases up to 3.60 times.
- The Bejan number (Be) graphs indicate that near the stretching surface, the effects of heat transfer and diffusion entropy are predominant, while in regions farther away, the entropy effects due to fluid friction and the magnetic field become more significant.
- At the surface of the plate, the average Bejan number decreased up to 3.21 times as the magnetic parameter varied from 0.1 to 1.1.

The current approach towards the entropy analysis can further be extended to the problem related to Casson nanofluids in the presence of a magnetic field.

## Figures and Tables

**Table 1:** List of numerical values for skin friction coefficient and local Nusselt and Sherwood numbers using γ=0.9, Bi=0.9 and $R_{e}=0.7$.

Pr	${Gr}_{L}$	${Gr}_{C}$	β	Sc	$R_{d}$	ϵ	${2^{-\frac{1}{2}}Re}_{x}^{\frac{1}{2}}C_{fx}$	$\sqrt{2}{Re}_{x}^{-\frac{1}{2}}{Nu}_{x}$	$\sqrt{2}{Re}_{x}^{-\frac{1}{2}}{Sh}_{x}$
0.7							1.5126	0.5854	1.2851
1.0							1.4793	0.6245	1.2841
	0.4						1.3716	0.6225	1.2819
	0.6						1.5862	0.6264	1.2863
		0.5					1.3819	0.6227	1.2824
		0.6					1.4793	0.6245	1.2841
			0.6				1.5691	0.6188	1.2798
			0.7				1.5307	0.6207	1.2814
				0.4			1.5759	0.6222	1.1487
				0.6			1.4947	0.6196	1.4012
					0.6		1.5101	0.6509	1.4016
					0.7		1.5245	0.6814	1.4020
						0.9	1.4947	0.6196	1.4012
						1.1	0.5685	0.6388	1.4402

**Table 2:** Average entropy number ($\bar{N_{s}})$ and average Bejan number $\left(\bar{Be}\right)$ with variation of Br at fixed $\gamma =0.3,Pr=0.7,Sc=0.9,R_{d}=0.3,{Gr}_{c}=0.8,{Gr}_{l}=0.5,\beta =1.0,Bi=0.1,\varepsilon =0.1,Re=0.7,\Omega_{c}=\Omega_{t}=0.1,M=0.1$.

Br	0.1	0.3	0.5	0.7	0.9	1.1
$\bar{N_{s}}$	0.0753	0.2174	0.3594	0.5015	0.6435	0.7856
$\bar{Be}$	0.0891	0.0563	0.0423	0.0341	0.0287	0.0247

**Table 3:** Average entropy number ($\bar{N_{s}})$ and average Bejan number $\left(\bar{Be}\right)$ with variation of β at fixed $\gamma =0.3,Pr=0.7,Sc=0.9,R_{d}=0.3,{Gr}_{c}=0.8,{Gr}_{l}=0.5,Br=0.1,Bi=0.1,\varepsilon =0.1,Re=0.7,\Omega_{c}=\Omega_{t}=0.1,M=0.1$.

β	1	2	3	4	5	6
$\bar{N_{s}}$	0.0753	0.0732	0.0724	0.0719	0.0717	0.0715
$\bar{Be}$	0.0891	0.0881	0.0877	0.0875	0.0873	0.0872

**Table 4:** Average entropy number ($\bar{N_{s}})$ and average Bejan number $\left(\bar{Be}\right)$ with variation of M at fixed $\gamma =0.3,Pr=0.7,Sc=0.9,R_{d}=0.3,{Gr}_{c}=0.8,{Gr}_{l}=0.5,Br=0.1,Bi=0.1,\varepsilon =0.1,Re=0.7,\Omega_{c}=\Omega_{t}=0.1,Br=0.1$.

M	0.1	0.3	0.5	0.7	0.9	1.1
$\bar{N_{s}}$	0.0753	0.1327	0.1901	0.2475	0.3049	0.3623
$\bar{Be}$	0.0891	0.0584	0.0452	0.0372	0.0317	0.0277

**Table 5:** Average entropy number ($\bar{N_{s}})$ and average Bejan number $\left(\bar{Be}\right)$ with variation of $R_{d}$ at fixed $\gamma =0.3,Pr=0.7,Sc=0.9,Br=0.1,{Gr}_{c}=0.8,{Gr}_{l}=0.5,Br=0.1,Bi=0.1,\varepsilon =0.1,Re=0.7,\Omega_{c}=\Omega_{t}=0.1,M=0.1$.

$R_{d}$	0.5	2.5	4.5	6.5	8.5	10.5
$\bar{N_{s}}$	0.0753	0.0826	0.0878	0.0925	0.0968	0.1010
$\bar{Be}$	0.0891	0.1079	0.1296	0.1500	0.1682	0.1841

## Data Availability

The original contributions presented in the study are included in the article, further inquiries can be directed to the corresponding author.
